# Nebulized Bacterioruberin/Astaxanthin-Loaded Nanovesicles: Antitumoral Activity and Beyond

**DOI:** 10.3390/ijms26178607

**Published:** 2025-09-04

**Authors:** Victoria Rebeca Dana González Epelboim, Diego G. Lamas, Cristián Huck-Iriart, Ezequiel Nicolas Caputo, Maria Julia Altube, Horacio Emanuel Jerez, Yamila Roxana Simioni, Kajal Ghosal, Maria Jose Morilla, Leticia Herminia Higa, Eder Lilia Romero

**Affiliations:** 1 Nanomedicine Research and Development Centre (NARD), Science and Technology Department, National University of Quilmes, Roque Saenz Peña 352, Bernal 1876, Buenos Aires, Argentina; 2 Laboratorio de Cristalografía Aplicada (LCA), Instituto de Tecnologías Emergentes y Ciencias Aplicadas (ITECA), Escuela de Ciencia y Tecnología (ECyT), UNSAM-CONICET, Campus Miguelete, San Martín 1650, Buenos Aires, Argentina; 3 ALBA Synchrotron Light Source, Carrer de la Llum 2–26, Cerdanyola del Vallès, 08290 Barcelona, Spain; 4 Department of Pharmaceutical Technology, Jadavpur University, 188, Raja Subodh Chandra Mallick Rd., Jadavpur, Kolkata 700032, West Bengal, India

**Keywords:** lungs, inhalation, xanthophylls

## Abstract

The membranes of halophilic archaea are a source of novel biomaterials, mainly of isoprenoid nature, with therapeutic properties practically unraveled. Here, we explored the antitumoral activity of neutral archaeolipids (NAs, such as bacterioruberin, astaxanthin, and dihydrosqualene) present in the total archaeolipids (TAs) (a fraction from the first step of lipid extraction by the modified Blight and Dyer technique) extracted from halophilic archaea Halorubrum tebenquichense, and formulated as TA-nanoarchaeosomes (TA: polar archaeolipids (PAs): Tween 80, 5:5:4 *w*:*w*:*w*, TA-nanoARC). The structure of 300.3 ± 84.2 nm TA-nanoARC of 0.59 ± 0.12 polydispersity index and −20 ± 3.7 mV ζ potential as determined by SAXS modelling, revealed that NA reduced the hydrophobic core and enlarged its hydrophilic section in comparison to TA-lacking bilayers (nanoARC), while preserving the width (~50 Å) and unilamellarity. Stable to storage and nebulization, TA-nanoARC was cytotoxic on A549 cells after 48 h, with an IC50 expressed as [bacterioruberin] of 0.15 μg/mL (~0.20 µM), comparable to or lower than the IC50 of docetaxel or cisplatin. Such cytotoxicity was exerted at a concentration harmless to macrophages (mTHP-1 cells). Besides, the conditioned medium from TA-nanoARC nebulized on A549 cells reduced the expression of the CD204/SRA-1, an M2 phenotype marker, and induced pro-inflammatory activity, comparable to or to a greater extent than that induced by lipopolysaccharide, including IL-6 and TNF-α, in mTHP-1 as a model of tumor-associated macrophages. The endocytosis of TA-nanoARC by A549 cells induced Lysotracker red fluorescence to fade and blur. This suggested the internalization of the highly viscous and ordered TA-nanoARC rich in NAs and subsequent lysosomal dysfunction (and not its antioxidant activity), as responsible for the selective damage on A549 cells. These are the first results showing that nebulized TA-nanoARC, lethal to A549 cells and modulating mTHP-1 cell phenotype, may act as antitumorals in the absence of cytotoxic drugs.

## 1. Introduction

Lung cancer constitutes the most prevalent cancer diagnosis worldwide, and non-small cell lung cancer (NSCLC) accounts for nearly 85% of all lung cancer cases [1]. Chemotherapy is part of the complex treatment of NSCLC [2,3] and is carried out by administering intravenous cytotoxic agents such as cisplatin, docetaxel, or gemcitabine [4]. The primary limitations of NSCLC chemotherapy include suboptimal drug delivery efficiency, drug resistance, and toxic side effects [5]. Together with pleural mesothelioma, NSCLC is among the most lethal and therapy-resistant tumors in humans [6]. In this context, the search for new, more efficient, and less toxic treatments based on agents other than classical chemotherapeutics becomes relevant.

Aerosolization is the only non-invasive route for direct drug administration to the lung epithelium. By avoiding the gastrointestinal tract, the degradation of the active ingredient and first-pass metabolism in the liver are decreased [7]. Aerosolization of cytotoxic drugs may limit their systemic access and toxicity [8]. Moreover, the aerosolization of nanomedicines may constitute a promising strategy to treat lung cancers. Besides offering systemic evasion, the inhalation of nanoparticles provides magnified activity upon endocytosis by target cells such as epithelial, fibroblasts, and/or macrophages [9,10,11].

At the moment, however, only the nebulizable liposomal antimicrobial ARIKAYCE^®^ (Amikacin Liposome Inhalation Suspension) [12,13,14] has been approved in the USA (September 2018 [15]) and Europe (October 2020 [16]) for the treatment of refractory *Mycobacterium avium complex* (*MAC*) lung disease; aside from that, a liposomal formulation of inhalable ciprofloxacin is currently in phase 3 [17]. Their biocompatibility and regulatory acceptance highlight the suitability of liposomes for lung aerosolization [18]. The weakest point of liposomes, however, is their structural stability. Nebulizable liposomes must add cholesterol to their structure and employ synthetic or hydrogenated fully saturated phospholipids (PLs) to resist nebulization stress. There are, however, alternative lipids to classical phospholipids, which offer new and attractive structural properties, making them suitable for the manufacture of nanomedicines.

Polyisoprenoids, molecules derived from the isoprene unit (2-methyl-1,3-butadiene), for example, are widely distributed in nature, and are synthesized by organisms belonging to the three domains of life on Earth [19]. Among them, a group of polyisoprenoids with features much less explored than their relatives from the Eukarya and Bacteria domains, are those produced by microorganisms belonging to the Archaea domain, known as archaeolipids. Archaeolipids can be both polar and neutral [20]. The structure of the polar ones differs from that of classical phospholipid glycerol esters. Their lack of unsaturations, the replacement of esters by ethers, and their 2,3-chirality make refractory polar archaeolipids (PAs) resistant to chemical or enzymatic hydrolysis in lysosomes, or to undergo oxidative attack. In an aqueous medium, PAs form nanovesicles known as nanoarchaeosomes. Nanoarchaeosomes exhibit different structural characteristics (ultra deformability, pH-sensitivity), according to the molecules mixed with the PA matrix [21,22]; or the chemical modifications made on their polar heads [23,24]. Given their structural heterogeneity, we will refer to any nanovesicles containing archaeolipids in their composition as nanoarchaesomes. Unlike liposomes, nanoarchaeosome lamellae consist of PL polyisoprenoid diether bilayers or tetraether monolayers [25]. Initially, nanoarchaeosomes were used as adjuvant agents [26] and shortly afterward in drug delivery [27]. PAs of halophilic archaea are rich in PGP-Me, and nanoarchaeosomes prepared with these PAs are massively internalized by target cells expressing SRA1 [21]. Overall, nanoarchaeosomes not only offer greater structural resistance to both enzymatic and physicochemical aggressions than liposomes [28] but are also able to cope with the intense mechanical stresses of aerosolization [21].

Given the growing interest in carotenoids of natural origin, sustainable, and from extreme environments [29,30,31], among the neutral archaeolipids (NAs) synthesized by halophilic archaea, bacterioruberin (BR), a strange xanthophyll C50, is distinguished. BR is a powerful antioxidant agent, with demonstrated anti-inflammatory, antitumor, and antimicrobial activity [32,33]. Like astaxanthin (ATX) extracted from the algae *Haematococcus pluvialis*, BR uses are expected to have applications beyond dietary supplements and to be considered as a potential therapeutic agent [20,33,34,35]. Most reports on the in vitro activity of BR, however, have been made with BR dissolved in an organic solvent [36,37,38] except for a small number of publications where BR was formulated into the core of nanostructured lipid carriers (NLCs) [39,40] and in liposomes or in nanoarchaeosomes (whose PA matrix increased the structural stability of the BR) [41]. The aerosolization of properly formulated BR, for instance, would favor its bioavailability on the lung surface, maximizing its antimicrobial/anti-inflammatory activity.

Recently, unlike liposomes, nanoarchaeosomes made of PA from *H. tebenquichense* have been demonstrated to be bioactive, e.g., anti-endothelial inflammation [42]. More recently, large nanoarchaeosomes (too large to be widely endocytosed) resulted in cytotoxic to typically non-phagocytic A549 cells [43]. These reports suggest that nanoarchaeosomes may offer unraveled therapeutic properties, despite their pharmacodynamics remaining largely unknown. In such a context, we will explore the structure and antitumor activity of nanoarchaeosomes made of total archaeolipids (TAs) (TA-nanoARC), from the halophilic archaea *H. tebenquichense* and compare them with nanoARC, prepared only with PA. The TA results from the first step of lipid extraction of the modified Bligh and Dyer technique, prior to the separation of polar and neutral lipids by cold acetone precipitation. According to Kates 1995 [44], the TA may contain a high proportion and variety of NA, ranging from C20 (as geranylgeraniol), C30 (as squalene), C40 as lycopersene, or C50 (as BR and its isomers). Unlike the core of NLC, methyl perpendicular to the longitudinal axis of the carbon chain of PAs in archaeolipid bilayers, offers an environment similar to isostearic acid, strongly trapping small molecules difficult to solubilize, such as imiquimod (a class IV biopharmaceutical, poorly soluble in hydrophilic or lipophilic solvents and a poorly permeable drug) [45], tacrolimus and cannabidiols such as CBD (class II, poorly hydrosoluble, highly permeable drugs). This property, although it facilitates their suspension in an aqueous medium, can be a disadvantage for those molecules that must dissociate from the PA matrix to exert their effect. Formulated into TA-nanoARC, a high aqueous NA concentration could be achieved, which could also be nebulized. However, the NAs would remain strongly associated with PAs when delivered to the endo/lysosomes upon TA-nanoARC endocytosis. The effect of NAs formulated in this way on cell viability is unknown, and for the first time, we explore it on A549 cells as a model of NSCLC.

## 2. Results

### 2.1. Extraction and Characterization of Archaeolipids

**Figure 1:**
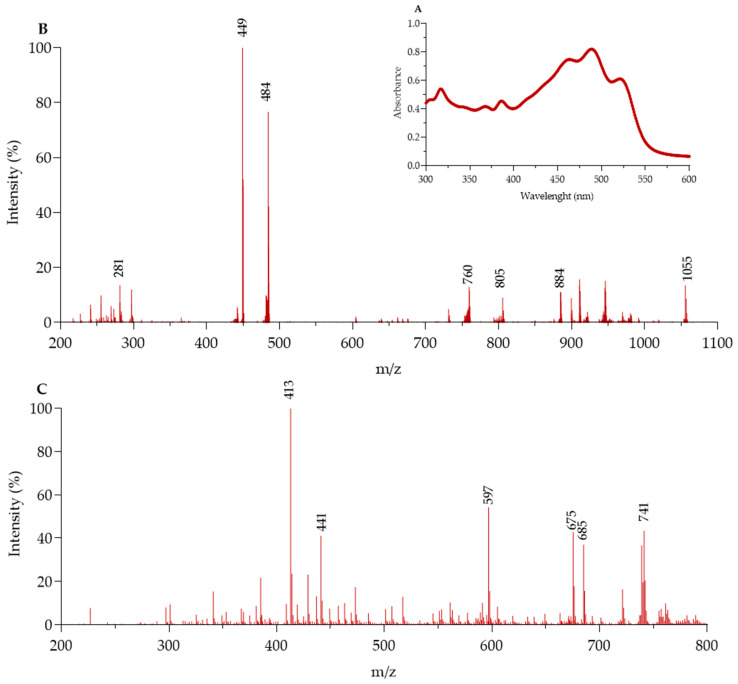
Characterization of total archaeolipids (TAs) from *H. tebenquichense*. (**A**) UV-vis spectra; (**B**) Electrospray ionization–mass spectrometry (ESI-MS) analysis of polar archaeolipids (PAs) in negative ion mode and (**C**) neutral archaeolipids (NAs) in positive ion mode.

The TAs extracted from the halophilic archaea *H. tebenquichense* contained 165 ± 42 mg of PLs and 1.5 ± 0.5 mg of BR/100 g of cell paste (*n* = 6). The PAs contained 177 ± 54 mg of PLs/100 g of cell paste (*n* = 8). The UV–visible spectrum of TAs dissolved in acetone revealed the typical three-finger peaks of the BR with maximums at 455, 490, and 525 nm, and the two peaks corresponding to the cis configurations, with maximums of wavelengths at 365 and 380 nm [46,47] (Figure 1A). In contrast, the spectrum of PA lacked peaks between 400 and 550 nm.

The ESI-MS spectrum in negative mode of TAs showed peaks at 281 (corresponding to a phytanyl chain [48], 449 (phosphatidylglycerophosphate methyl ester, PGP-Me, as a bicharged ion), 484 (loss of a phytanyl chain (280 Da) from cardiolipin bisphosphatidylglycerol, BPG), 760 (BPG, as a bicharged ion), 805 (phosphatidylglycerol, PG), and 1055 (sulfated mannosyl glucosyl diether (S-DGD)) *m*/*z* ratio in coincidence with previous reports [49] (Figure 1B). Additionally, a peak at 884 *m*/*z* ratio (phosphatidylglycerosulfate, PGS) previously identified in *H. salinarum* by [50], but not observed before in PA from *H. tebenquichense* [49], was detected.

Likewise, the ESI-MS spectrum in positive mode of the TA showed peaks of highest intensity at 413 ([dihydrosqualene-H]+), 597 ([astaxanthin-H]+ [51]), 675 (tetra anhydro BR), 685 (tris anhydro BR), and 741 ([BR-H]+ [52] *m*/*z*) (Figure 1C). Other peaks of lower intensity, such as 441 *m*/*z* [53], have been ascribed to stable fragment ions.

### 2.2. Structural Characterization of TA-nanoARC and nanoARC

The structural features of TA-nanoARC and nanoARC are shown in Table 1. Both nanoarchaeosomes were sub-micron-sized and exhibited negative and comparable ζ potentials. TA-nanoARC suspensions showed the typical red color of BR. Laurdan fluorescence showed that nanoARC bilayers (made of PA and Tween 80) were much more disordered and slightly more fluid (inversely correlated to fluorescence anisotropy) than the TA-nanoARC bilayers (made of PAs, Tween 80, and NA). These suggest that NA ordered and increased the microviscosity of TA-nanoARC bilayers.

**Figure 2:**
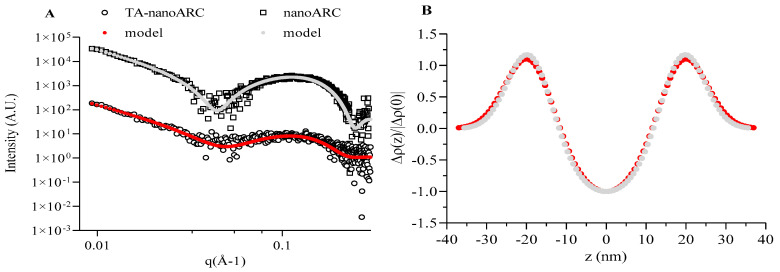
SAXS data of nanoARC and TA-nanoARC. (**A**) Log–log profiles with solid lines corresponding to model fits. (**B**) Electron density profiles derived from data fitting. *n* = 3.

Figure 2A displays SAXS data in a log-log scale for nanoARC and TA-nanoARC, along with the corresponding fits obtained using our model. Good agreement was found in both cases. All SAXS patterns showed similar features consistent with bilayer systems, specifically a broad bump around 0.1 Å^−1^ and the intensity decay with q^−2^ power law at low angles, characteristic of large 2D structures. Importantly, there was no indication of multilayer formation. The core (aqueous compartment), bilayer thickness, and hydrophilic layer were very similar for nanoARC and TA-nanoARC, with values close to 25 nm, 50 nm, and 13 nm, respectively (Table 2).

Figure 2B shows the electron density profiles derived from SAXS data fitting. While TA-nanoARC produced a smoother SAXS curve than nanoARC, the extracted structural parameters were largely similar. The key fitting variables were the amplitude and width of the Gaussian function used to describe the electron density in the hydrophilic region, with the parameters for the hydrophobic core held fixed to isolate the effects of processing or analyte loading.

**Figure 3:**
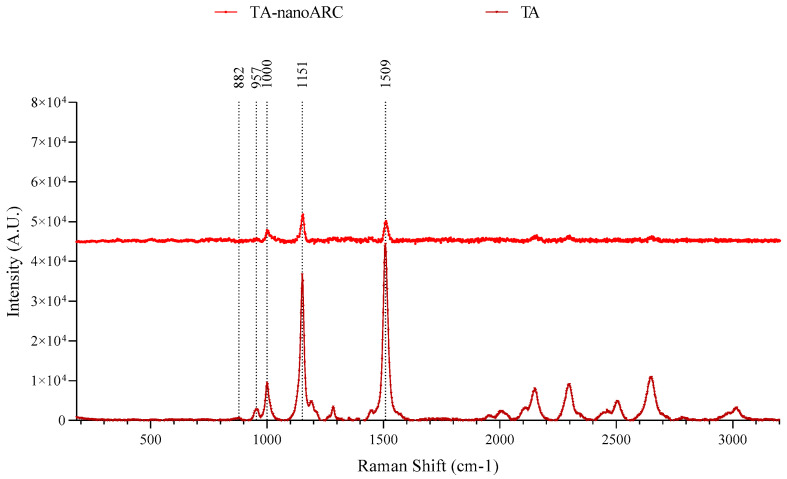
RAMAN spectra of TA-nanoARC and TA of *H. tebenquichense*.

The Raman spectrum of TA (Figure 3) showed BR at 1000 cm^−1^ (in-plane rocking modes of CH_3_ groups attached to the polyene chain coupled with C–C bonds); 1151 cm^−1^ (the stretching vibrations of C–C single bonds coupled with C–H in-plane bending modes) and 1509 cm^−1^ (C═C stretching band) signature peaks as reported by [41] for BR extract. The Raman spectrum of TA-nanoARC showed the same peaks, while nanoARC and Tween 80 showed no Raman signals.

**Figure 4:**
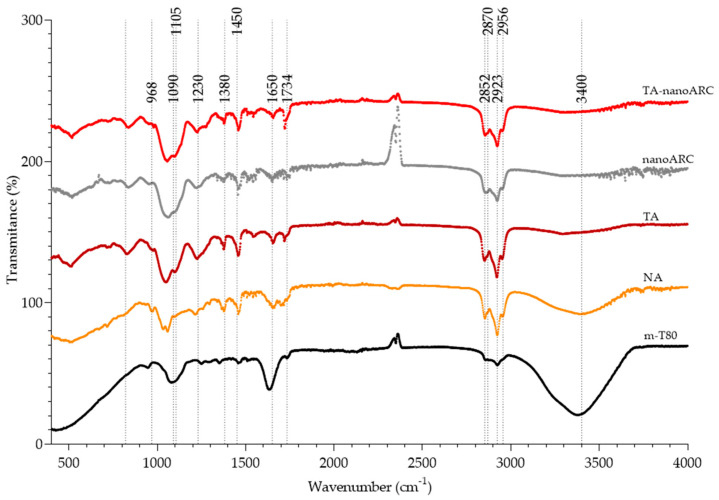
Attenuated total reflectance—Fourier transform infrared spectroscopy (ATR-FTIR) spectra of TA-nanoARC, nanoARC, Tween 80 micelles (m-T80), TA, and neutral archaeolipids (NAs) of *H. tebenquichense*.

The FTIR spectra of TA-nanoARC, nanoARC, TA, and NA were dominated by poly-isoprenoid chain signals, common to TA (rich in NA, such as BR) and PA (rich in PA, such as PGP-Me) (Figure 4). No significant modifications were observed in the peaks from PA shown in Table 3, except in the ether stretching bands, which in TA-nanoARC and nanoARC were shifted toward lower wavenumbers, suggesting the establishment of hydrogen bonds between the glycerol skeleton of PA and Tween 80. The disappearance of the broad OH stretching band from Tween 80 in TA-nanoARC can also be attributed to the establishment of hydrogen bonding between Tween 80 and NA.

### 2.3. Antioxidant Activity

**Figure 5:**
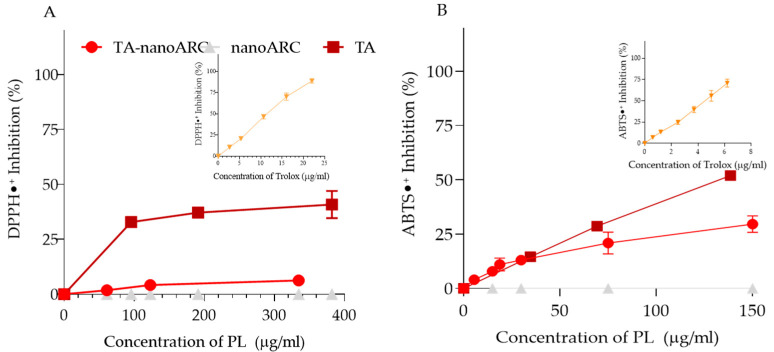
Antioxidant activity of TA-nanoARC, nanoARC, total archaeolipids (TAs), and Trolox. Scavenging of DPPH•^+^ (**A**,**B**) ABTS•^+^ radicals as a function of phospholipid (PL) or Trolox concentrations. Insets display Trolox as a standard antioxidant control. *n* = 3.

The ability of TA-nanoARC to reduce the DPPH radical was lower than that of TA, whereas nanoARC showed no activity (Figure 5A). These results were reasonable, since nanoARC lacks NA, and the content of BR in TA doubles the amount of BR in TA-nanoARC (9.43 vs. 4.4 μg BR/mg PL). Since the inhibitory activities of TA and TA-nanoARC were not linear with concentration, it was not possible to calculate an IC_50_ value. However, between 0.94 and 3.6 μg BR/mL, TA reduced by 35–40% the DPPH radical. In contrast, at 0.94 μg BR/mL, the antioxidant activity of TA-nanoARC was seven times lower. Such a difference suggests that BR in TA was oxidized along with TA-nanoARC manufacture. The inhibitory activity of Trolox was linear with concentration (see inset in Figure 5A), with an IC_50_ of 10.5 μg Trolox/mL. The IC_50_ of TA was 17.7 μg BR/mL, slightly higher than the 13.5 μg BR/mL previously reported for BR extract [41].

The inhibitory activity of the ABTS radical by nanoARC was also zero. Again, the inhibitory activity of TA-nanoARC was not linear with concentration (Figure 5B). However, the corresponding IC_50_ of TA and TA-nanoARC were 1.25 and 6.8 μg BR/mL, respectively. In this case, the activity of TA was 5.4 times greater than that of TA-nanoARC, restating the idea that BR would be oxidized during TA-nanoARC manufacture. The inhibitory activity of Trolox was linear with concentration, with IC_50_ of 4.3 μg Trolox/mL (see inset in Figure 5B).

### 2.4. Stability Storage

**Figure 6:**
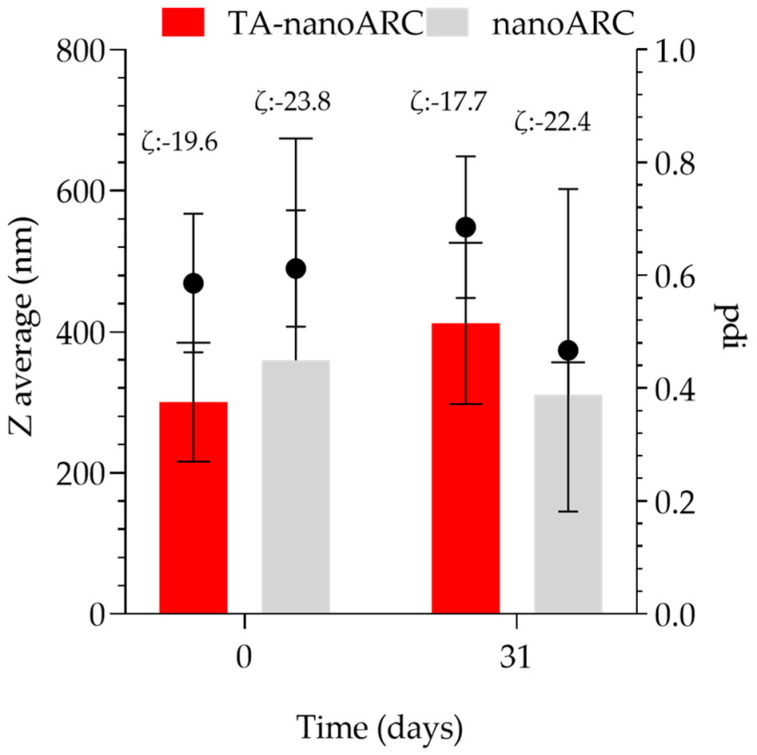
Colloidal stability under storage of TA-nanoARC and nanoARC. The statistical analysis indicating no significant difference was determined using Wilcoxon signed-rank test. *n* = 3.

Both TA-nanoARC and nanoARC were stable in terms of size, polydispersity, and ζ potential after 31 days (Figure 6).

### 2.5. Stability to Nebulization

Size, polydispersity, ζ potential, and PL recovery were determined before and after nebulization of TA-nanoARC and nanoARC. PL recovery remained relatively high after nebulization for both formulations. However, size increased particularly in nanoARC (306 nm to 376 nm), indicating a mild aggregation tendency. The polydispersity also showed an increase in TA-nanoARC, reflecting a broader size distribution. In contrast, nanoARC showed a slight improvement. ζ potential values were similar for both formulations. Overall, both nanoARC and TA-nanoARC exhibited optimal stability after nebulization (Table 4).

### 2.6. Cytotoxicity over Nebulized A549 and mTHP-1

**Figure 7:**
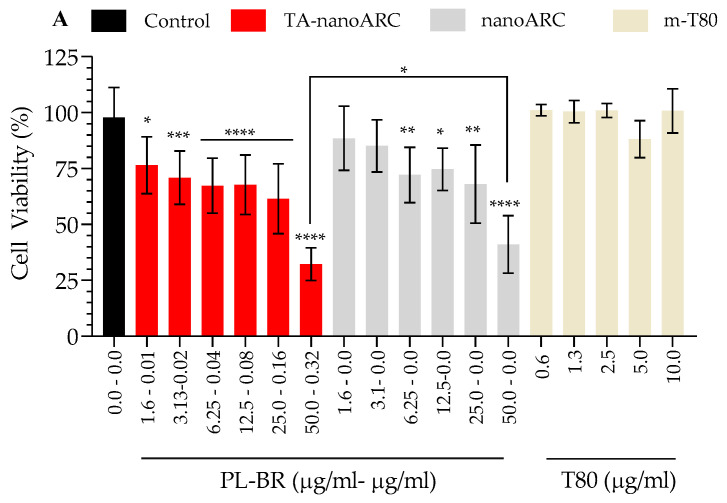

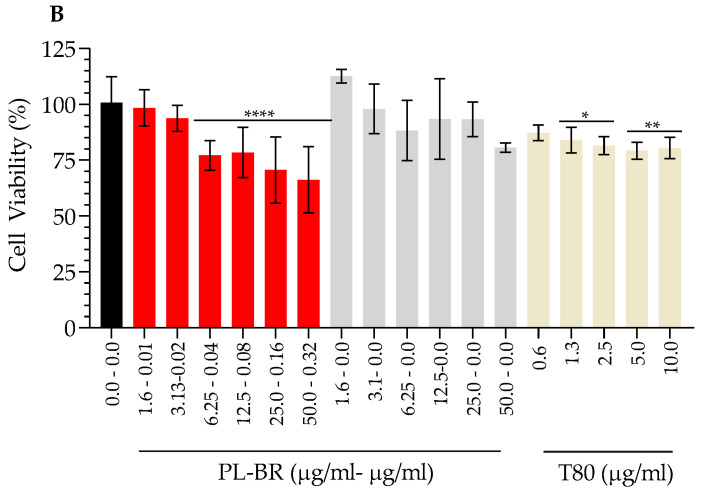
Cytotoxicity of A549 cells (**A**) and mTHP-1 (**B**) upon 48 h incubation with TA-nanoARC, nanoARC, or m-T80 after nebulization, expressed as function of phospholipid (PL) and bacterioruberin (BR) or Tween 80 (T80) concentration, *n* = 3. The control group consisted of cells cultured in medium only. Statistical significance compared to control was determined using a one-way ANOVA followed by Dunnett’s test, * *p* < 0.05; ** *p* < 0.01,*** *p* < 0.001, **** *p* < 0.0001.

Figure 7 shows the effect of the formulations on the viability of A549 cells after 48 h. IC_50_ TA-nanoARC was 33.8 μg PL/mL (0.15 μgBR/mL), while the IC_50_ of nanoARC was ~50 μg PL/mL. TA-nanoARC was therefore ~1.5 times more cytotoxic than nanoARC. This difference can be attributed to the presence of NA in TA-nanoARC bilayers (Figure 7A, Table 5).

Cytotoxicity on epithelial cells of the respiratory tract may be caused by inhalation of Tween 80 present in TA-nanoARC or nanoARC. However, Tween 80 micelles (m-T80) at a concentration comparable to those in nanoarchaeosomes did not cause cytotoxicity on A549 cells. Moreover, at the IC_50_ of TA-nanoARC on A549 cells, the Tween 80 concentration was ~14 μg per/mL, about ~500 times lower than that reported harmless on human bronchial epithelial cells BEAS-2B [54]. At slightly lower doses (10 µg/mL), Tween 80 would not induce pulmonary surfactant inactivation [55].

The nanoarchaeosomes, on the other hand, were less cytotoxic on mTHP1: the IC_50_ of TA-nanoARC and of nanoARC were ~65 μg PL/mL, and 210 μg PL/mL, respectively (Figure 7B, Table 5). The cytotoxicity on mTHP-1 followed the same trend as observed on A549 cells: the presence of NA in the bilayers decreased cell viability. Nonetheless, the cytotoxicity on A 549 cells of TA-nanoARC was ~twice that over THP-1.

### 2.7. Intracellular ROS in A549 and mTHP-1

**Figure 8:**
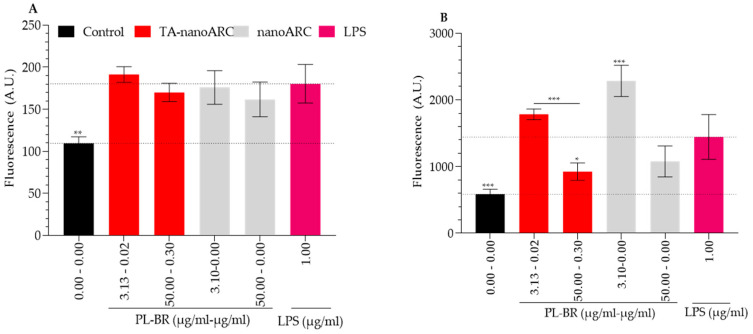
Antioxidant activity on A549 cells (**A**) and mTHP-1 (**B**) upon 24 h incubation with TA-nanoARC or nanoARC. Statistical significance compared to control was determined using a one-way ANOVA followed by Dunnett’s test, * *p* < 0.05; ** *p* < 0.01, *** *p* < 0.001. *n* = 3.

Neither TA-nanoARC nor nanoARC modified the minimal ROS induced by LPS in A549 cells compared to that produced in mTHP-1 (Figure 8A). Instead, both formulations (despite nanoARC containing no antioxidants) at 50 µg PL/mL, reduced the intracellular ROS in mTHP-1 (Figure 8B).

### 2.8. Effect on Mitochondrial Membrane Potential and Lysosomes of A549 Cells

**Figure 9:**
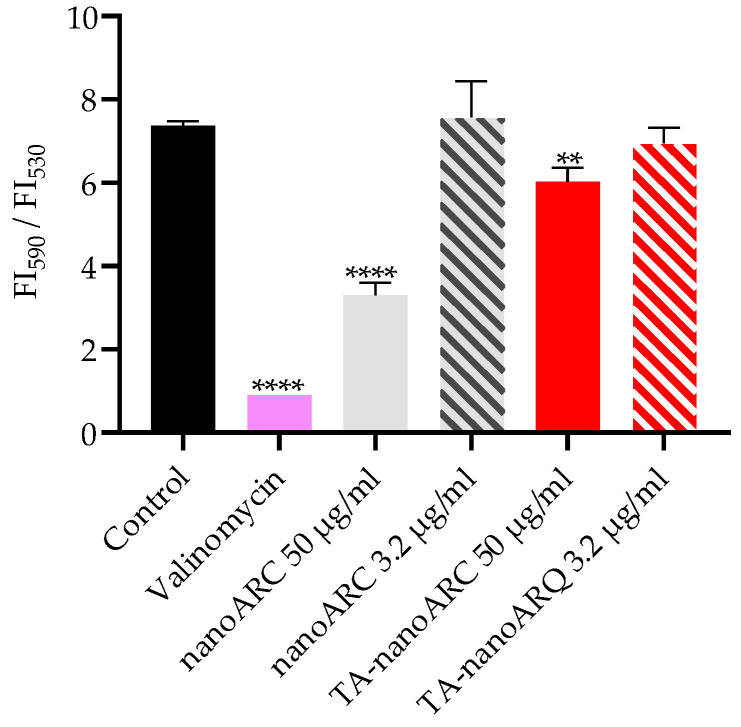
Mitochondrial membrane potential of A549 cells upon 24 h incubation with TA-nanoARC or nanoARC. Statistical significance compared to control was determined using a one-way ANOVA followed by Dunnett’s test, ** *p* < 0.01, **** *p* < 0.0001.

At 50 µg PL/mL, TA-nanoARC barely reduced the mitochondrial membrane potential, whereas nanoARC strongly reduced it, as shown in Figure 9.

**Figure 10:**
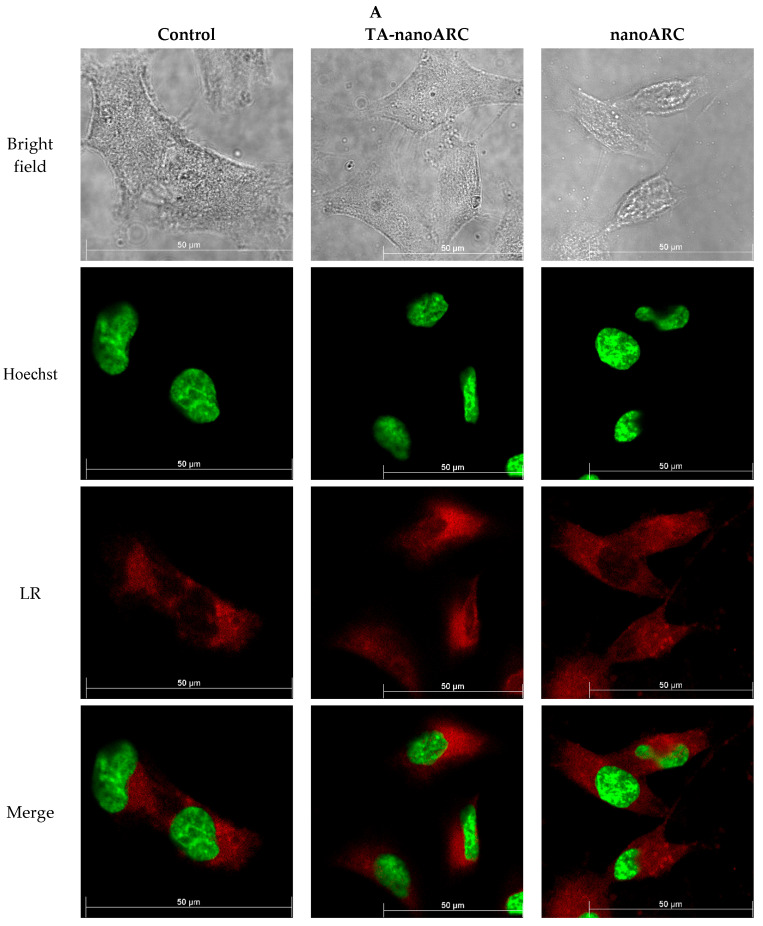

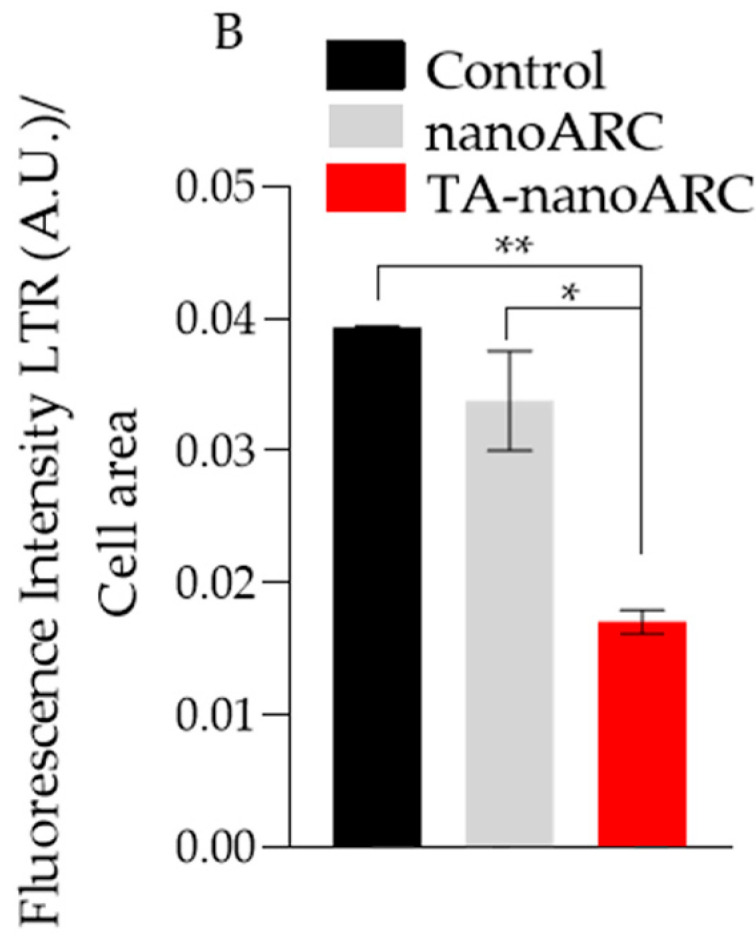
(**A**) Confocal fluorescence images of A549 stained with Lysotracker (red, LR) and Hoechst (green) after incubation with medium, TA-nanoARC, or nanoARC. Magnification 63× (**B**) Lysotracker Red (LR) fluorescence intensity inside the cells per cell was quantified using Image J Significant differences between formulations were determined using a one-way ANOVA followed by Dunnett’s test, * *p* < 0.05; ** *p* < 0.01.

In Figure 10 it is shown that nanoARC caused no visible structural perturbation of lysosomes’ fluorescence, compared with the untreated control, except for the appearance of bright and small vesicles inside and outside the cells. TA-nanoARC, on the other hand, reduced the fluorescence intensity of the dye and blurred it over the cell cytoplasm.

### 2.9. Response of mTHP-1 to Conditioned Media of A549 Cells

#### 2.9.1. Immunomodulation of mTHP-1

**Figure 11:**
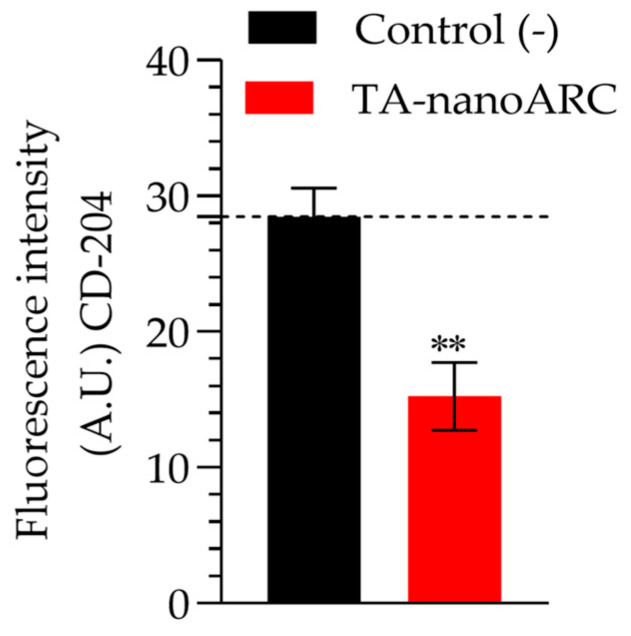
CD-204 fluorescence intensity on mTHP-1 cells incubated with A549 cell-conditioned media at 6.3 µg/mL phospholipids (PLs) of TA-nanoARC or nanoARC. Significance considered at ** *p* < 0.01.

Only the conditioned medium of A549 cells treated with TA-nanoARC was able to reduce the expression of CD204 (a marker of SRA-I) in mTHP-1, as shown in Figure 11.

#### 2.9.2. Pro-Inflammatory Cytokines from mTHP-1

**Figure 12:**
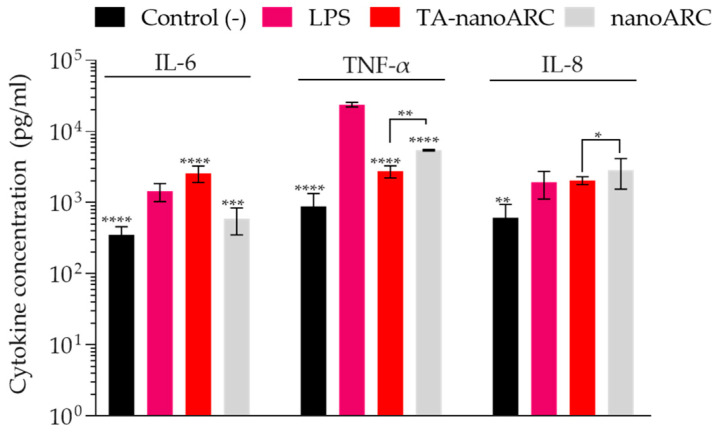
Proinflammatory cytokine production (IL-6, IL-8, and TNF-α) by mTHP-1 in response to conditioned media from A549 cells treated with TA-nanoARC, nanoARC, or culture medium as a negative control. LPS was used as a positive control. Treatment concentrations (µg/mL PL—µg/mL BR): Control: 0.00–0.00; TA-nanoARC: 6.30–0.04; nanoARC: 6.30–0.00; LPS (1 µg/mL). Statistical significance was assessed against LPS and between TA-nanoARC and nanoARC using a one-way ANOVA followed by Dunnett’s test, significance considered at * *p* < 0.05; ** *p* < 0.01 *** *p* < 0.001, **** *p* < 0.0001.

The conditioned medium of A549 cells treated with TA-nanoARC induced IL-6 to a higher extent than LPS; a lower amount of TNF-α, and no IL-8. The conditioned medium from nanoARC only induced TNF-α to an extent comparable to TA-nanoARC, as shown in Figure 12.

## 3. Discussion

Here we showed that TA-nanoARC (containing PA and xanthophylls such as the C50 bacterioruberin, the C40 astaxanthin, and dihydrosqualene) was cytotoxic on A549 cells after 48 h: its IC50 expressed as [BR from NA, formulated as TA-nanoARC] was 0.15 μg BR/mL (~0.20 µM). Such concentration is below the IC50 on A549 cells of drugs classically used to treat NSCLC: docetaxel (IC50 after 48 h: 0.24 µg/mL (0.3 µM)) [56]; cisplatin (IC50 after 48 h: ~9.3 µg/mL (~31 µM)) [57,58]; and paclitaxel (IC50 on A549 cells after 48 h: 1.64 µg/mL (~1.92 µM) [59]. Such cytotoxicity was exerted at concentrations harmless to THP-1 cells. The NA in TA-nanoARC increased cytotoxicity on A549 cells by 50% compared to nanoARC, indicating that if formulated as TA-nanoARC, a low concentration of NA may display a high antitumor activity. The following results will be briefly discussed, highlighting those that may explain the origin of the cytotoxicity of TA-nanoARC on A549 cells.

The first point is regarding the TA-nanoARC structure and composition. The ESI-MS spectrum in positive mode of TA showed the presence of the NA C_30_ dihydrosqualene (the partially saturated form of squalene) and C_50_ BR. In addition, the peak at 597 *m*/*z* suggested the presence of AXT, a C_40_ xanthophyll with 11 conjugated bonds and a topological polar surface area of 76.4 Å^2^ [60]. AXT is typically extracted from the algae *Haematococcus pluvialis*, has important commercial relevance as a strong antioxidant and as an antitumor. Although its production has been described by archaea from the *Haloferacaceae* family, here it is reported for the first time in a member of the *Halorubraceae* family. BR offers comparable or higher antioxidant activity and higher polarity than AXT, and exhibits unique structural characteristics, including a long chain of 13 conjugated double bonds [61], four hydroxyl groups located at the terminal positions, and a topological polar surface area of 80.9 Å^2^ [62,63]. The SAXS modelling suggested that these NAs are accommodated in the PA matrix of TA-nanoARC, reducing its hydrophobic core and enlarging its hydrophilic section compared to nanoARC, while preserving the unilamellarity.

To facilitate the hydration of the TA film, the lipids were added with the polysorbate non-ionic detergent Tween 80 (a P-gp efflux inhibitor at 15 µM (concentration comparable to that in nanoarchaeosomes) [64]. NanoARC included the same proportion of Tween 80 to make it comparable with TA-nanoARC. Both nanoarchaeosomes, despite the high Tween 80 content, showed acceptable structural stability over a short period of storage in darkness and 4 °C, maintaining their colloidal structure, undergoing nebulization stress without altering their population size or ζ potential.

The second point involves the source/mechanism of cytotoxicity of TA-nanoARC on A549 cells. A reduction of MMP could explain its detrimental effect [65]. However, only 50 µg PL/mL nanoARC reduced the MMP of A549 cells, while at the same concentration of PLs, TA-nanoARC slightly affected the MMP. 

Another cause of cell death could be a lysosomal dysfunction subsequent to material overloading upon nanoparticles endocytosis [66]. Human and mouse macrophages, for example, extensively internalize nanoarchaeosomes made of PAs of *H. tebenquichense* [21,67] due to their high content in PGP-Me, a ligand of SRA1. SRA1 is expressed primarily in macrophages, and mediates the intense internalization of different types of anionic ligands (heat shock proteins, amyloid-β (Aβ) surface molecules of Gram-negative and Gram-positive bacteria, and acetylated and oxidized low-density lipoprotein (OxLDL) [68,69,70,71] including nanoarchaeosomes from *H. tebenquichense* [21]). A549 cells, however, do not express SRA1 [72], and as previously reported [21], internalize nanoarchaeosomes much less extensively than macrophages. Therefore, a massive internalization of nanovesicles may not be responsible for the differential cytotoxicity between A549 cells and macrophages. Despite that, the cytotoxicity of TA-nanoARC and nanoARC on A549 cells was strikingly high. Besides being poorly internalized, nanoarchaeosomes were unable to reduce the modest amount of intracellular ROS induced by LPS after 24 h in A549 cells. After the same period, however, TA-nanoARC reduced the high oxidative stress generated by LPS in THP-1, confirming the antioxidant capacity of NAs. The fluorescence pattern of Lysotracker induced by nanoARC on A549 cells showed small, bright vesicles, suggesting highly acidic vesicles, probably of autophagic origin [73]. The finding is in line with those described in [74], where acutely damaged mitochondria stimulate lysosomal biogenesis and autophagy induction, as suggested by images of nanoARC endocytosis in Figure 10. Such a pattern completely differed from that induced by TA-nanoARC, where the cytoplasm was filled with blurred and diffuse fluorescence, indicative of loss of lysosomal acidity and potential dysfunction [75,76].

In sum, the events triggered in mitochondria and lysosomes of A549 cells after 24 h suggested two facts: one, that NAs within TA-nanoARC reversed the mitochondrial perturbation induced by nanoARC. The other, that the NAs themselves, would be responsible for the lysosomal dysfunction induced by TA-nanoARC, probably by making the bilayers more ordered and much less fluid, factors that may impede their processing by the lysosomal machinery [77]. Such dysfunction would also be more effective in reducing the viability of A549 cells than the reduction of MMP caused by nanoARC.

Finally, macrophages make up about half of the mass of the tumor microenvironment [78]. There, the tumor-associated macrophages (TAMs) acquire the M2 phenotype corresponding to alternatively activated macrophages, characterized by having high levels of CD169, CD206, and CD204 (or SR1A) [79] and promote the initiation and progression of NSCLC [80] by secreting various anti-inflammatory cytokines, activating signaling pathways, and interacting with other immune cells [81]. The regulation of M1/M2 polarization has recently been proposed as an anti-tumor therapeutic strategy, given the great plasticity of TAMs [82]. In this context, we determined the effect of conditioned media of A549 cells after nebulization with both formulations, on mTHP-1 as a model of TAM [83]. We observed that the conditioned medium from TA-nanoARC induced in mTHP-1 a reduction in the expression of the CD204/SRA-1 marker, typical of the M2 phenotype. The conditioned media of both formulations generated pro-inflammatory activity in mTHP-1, comparable to or to a greater extent than that induced by LPS. Only the conditioned medium of TA-nanoARC produced an intense secretion of IL-6, a cytokine whose role in immunity is context-dependent [84], initially described as a pro-tumor agent [85], more recently found responsible for orchestrating the action of different anti-tumor effectors [86,87].

Since a lower but consistent selective cytotoxicity on A549 cells was also observed for nanoARC (a formulation lacking NA), further in vivo studies will be needed to assess the true therapeutic impact of nanoarchaeosomes made of both polar and neutral archaeolipids on malignant cells. Our present results showed that the NA content in nebulized TA-nanoARC, not only increased their cytotoxicity on A549 cells but could also reprogram TAMs towards an anti-tumor phenotype. In the absence of classical cytotoxic drugs, an early lysosomal dysfunction is presumably involved in the higher and selective cytotoxicity of TA-nanoARC on A549 cells. 

## 4. Materials and Methods

### 4.1. Materials

6-Hydroxy-2,5,7,8-tetramethylchroman-2-carboxylic acid (Trolox), 2,2′-azino-bis(3-ethylbenzothiazoline-6-sulfonic acid) (ABTS), methylthiazolyltetrazolium bromide (MTT), β-mercaptoethanol, phorbol 12-myristate 13-acetate (PMA), and 2,2-diphenyl-1-picrylhydrazyl (DPPH), 6-lauroyl-2-dimethylaminonaphthalene Laurdan, JC-1 mitochondrial staining kit and lipopolysaccharides from *Escherichia coli* 0111:B4 (LPS) were obtained from Sigma-Aldrich, Argentina. Sodium pyruvate, L-glutamine, penicillin, streptomycin sulfate, trypsin, Minimal Essential Medium (MEM), and Roswell Park Memorial Institute (RPMI) medium were supplied by Gibco, Buenos Aires, Argentina. Human anti-CD204 monoclonal antibody, Hoechst 33342 (Hoechst), CM-H2DCFDA, and LysoTracker™ Red DND-99 were purchased from Thermo Fisher Scientific (Massachusetts, USA). Fetal bovine serum (FBS) was sourced from Internegocios Córdoba, Argentina. Tween 80 was provided by Biopack. All other reagents were of analytical grade and were acquired from Anedra, Argentina.

### 4.2. Growth of Archaeas and Lipid Extraction

Halophilic archaea *H. tebenquichense* were cultured in halophile-specific medium [88]. The cultures were grown in a homemade 25 L stainless-steel bioreactor at 40 °C with continuous agitation at 600 rpm. After 96 h of incubation, cells were harvested by centrifugation at 30,100× *g* for 10 min and stored as cell pastes at 4 °C. Total archaeolipids (TAs) were extracted from the cell paste using a modified Bligh and Dyer method adapted for extreme halophiles [44]. Briefly, 100 g of cell paste were suspended in 160 mL of an aqueous solution containing 20% *w*/*v* NaCl, 0.4% *w*/*v* KCl, and 2% *w*/*v* MgSO_4_, followed by the addition of 600 mL of a CHCl_3_:CH_3_OH mixture (1:2, *v*/*v*). The suspension was magnetically stirred for 2 h at room temperature to lyse the cells. The mixture was then centrifuged at 600× *g* for 5 min, and the pellet containing cellular debris was discarded. The supernatant was subjected to successive extractions with 60 mL of CHCl_3_:CH_3_OH:H_2_O (1:1:0.9, *v*/*v*) until the orange coloration of the upper aqueous phase disappeared. The TA fraction was recovered from the lower organic phase. The TA fraction was subsequently treated with cold acetone at a 1:9 (*v*/*v*) ratio (TA: acetone), resulting in the precipitation of polar archaeolipids (PsA), while the neutral archaeolipids (NAs) remained in solution [44]. Organic solvents from both TA and NA fractions were evaporated under reduced pressure using a rotary evaporator at 100 rpm and 37 °C. The PA and NA fractions were further dried under vacuum in a desiccator until a constant weight was reached and stored at −20 °C until use. PL (PL) content in both TA and PA fractions was quantified using the colorimetric microassay [89]. The NA fraction was characterized by UV–visible spectroscopy in the 300–700 nm range, and the concentration of bacterioruberin (BR) was estimated by absorbance at 490 nm using an average extinction coefficient of 2660 mL·mg^−1^·cm^−1^ [90].

### 4.3. Electrospray Ionization Mass Spectrometry (ESI-MS)

TA were analyzed by ESI-MS in negative ion mode for PA identification and in positive-ion mode for NA identification. Briefly, ESI-MS analyses were performed using a Bruker micrOTOF-QII mass spectrometer equipped with an electrospray ionization source. Samples were introduced via a loop injection system, using TA solutions prepared in CHCl_3_:CH_3_OH (1:1, *v*/*v*) for negative mode, and in CH_3_OH:HCOOH (1:1, *v*/*v*) for positive mode.

A volume of 5 µL was injected using a 10 µL loop and transferred to the electrospray ionization interface at a flow rate of 10 µL/min. The ion source parameters were as follows: nebulizing gas (air) at 4 L/min, drying gas (N_2_) at 4 L/min, capillary voltage at 4 kV, and mass range acquisition from 50 to 2000 *m*/*z* for both negative and positive ion modes.

### 4.4. Preparation of Nanovesicles

Total archaeolipids-nanoarchaeosomes (TA-nanoARC; PA:TA:T80 at a 1:1:0.8 *w*/*w*/*w* ratio) and nanoarchaeosomes (nanoARC; PA:T80 at a 2:0.8 *w*/*w* ratio) were prepared using the lipid film hydration method. Briefly, 5 mg of PA and 5 mg of TA (containing 45 μg of BR) or 10 mg of PA and 4 mg of T80 were dissolved in CHCl_3_:CH_3_OH (1:1, *v*/*v*) and transferred to round-bottom U-shaped microcentrifuge tubes for the preparation of TA-nanoARC and nanoARC, respectively. Organic solvents were evaporated under a N_2_ stream until a thin lipid film was formed. Subsequently, the lipid films were hydrated with 1 mL of 10 mM Tris, pH 7.4, containing 0.9% *w*/*v* NaCl (Tris-HCl buffer) under mechanical stirring, resulting in a final lipid concentration of 10 mg/mL. Particle size and lamellarity were reduced by sonication in a bath sonicator at room temperature (20 °C) for 60 min at 80 W of ultrasonic power and 40 kHz of frequency. As a control, micelles composed of Tween 80 at a concentration of 4 mg/mL (m-T80) were also prepared.

### 4.5. Characterization of Nanovesicles

Size and ζ potential: particle size and ζ potential were determined by dynamic light scattering (DLS) and phase analysis light scattering (PALS), respectively, using a NanoZsizer (Malvern Instruments, Malvern, United Kingdom). Samples were diluted 1:20 *v*/*v* in Tris-HCl buffer (50 μL of nanovesicles in 950 μL of Tris-HCl buffer) prior to measurement.

Phospholipid and BR quantification: PLs were quantified using the colorimetric phosphate microassay [89]. BR content was determined by measuring absorbance at 490 nm, applying an average mass extinction coefficient of 2660 mL·mg^−1^·cm^−1^ [90].

Raman spectroscopy: Raman spectra were acquired using i-Raman spectrometers (BWS415-532 and BWS465-785S, BWTEK, Plainsboro, NJ, USA) equipped with a Video Microscope (model BAC151B) at the Instituto de Investigaciones Fisicoquímicas Teóricas y Aplicadas (INIFTA), Buenos Aires, Argentina. Excitation was performed at 532 nm with acquisition times ranging from 500 to 1000 ms (2 or 5 accumulations), and laser power was set between 25 and 80%. Spectra were recorded over the 182–3200 cm^−1^ region.

Fourier Transform Infrared Spectroscopy with Attenuated Total Reflectance (ATR-FTIR): FTIR spectra were obtained using an Affinity-1 spectrometer (Shimadzu Co., Kyoto, Japan) at the Laboratorio de Obtención, Modificación, caracterización y evaluación de Materiales, LOMCEM, Universidad Nacional de Quilmes, Argentina. Briefly, 20 µL of each sample was deposited onto a diamond ATR module (GladiATR, Pike Technologies, Madison, WI, USA) and air-dried at room temperature (20 °C) for 30 min. Spectra were recorded with 100 scans in the 400–4000 cm^−1^ range at a resolution of 4.0 cm^−1^.

Small-Angle X-ray Scattering (SAXS): SAXS measurements were carried out using a XENOCS Xeuss 2.0 UHR HP200 SAXS-USAXS/WAXS instrument (Laboratorio de Cristalografía Aplicada, ITECA Institute, CONICET-UNSAM, San Martín, Argentina). It is equipped with a GeniX3D Cu Ultra Low Divergence microfocus source (Cu-Ka X-ray beam, average wavelength λ = 0.15419 nm). SAXS measurements were performed using a Dectris Pilatus3 R 200K-A hybrid pixel photon counting detector. The sample-to-detector distance was set at 1200 mm, resulting in a scattering vector (q) range of 0.085–3.50 nm^−1^. These q-values were calibrated using a silver behenate standard. All the experiments were performed in transmission geometry, using a high-flux configuration (beam size of 800 μm × 800 μm).

The SAXS normalized patterns were fitted using an in-house written program. The system was modelled as a planar bilayer of archaeolipids, exhibiting a distinct three-layer electron-density profile: a hydrophobic core (formed by ether-linked branched hydrocarbon chains), sandwiched between two dense hydrophilic layers corresponding to the polar headgroups. In agreement with previous models [91], the layers were described by Gaussian functions, $Gauss\left(r,r_{c},\sigma \right)=exp\left(-({r-r_{c})}^{2}/(2\sigma^{2})\right),$ to have smooth variation of the electron density as a function:

(1) $$∆\rho \left(r\right)=Sc_{ph}Gauss\left(r-R_{ph},\sigma_{ph}\right)+Sc_{hc}Gauss\left(r,\sigma_{hc}\right)+Sc_{ph}Gauss\left(r,R_{ph},\sigma_{ph}\right)$$

where the subscript ph refers to headgroups, hc to hydrocarbon. The Gaussian representing the hydrocarbon core had a fixed scale $Sc_{hc}=-1$.

For the actual calculation of the real amplitude factor A_p_(q), a general expression for centrosymmetric structures was used, as described by Pedersen 1997 [92].

(2) $$A_{P}\left(q\right)=\rho_{0}V(r_{0})A(q,r_{0})+\sum_{i=1}^{N}\left({\rho (r}_{i})-{\rho (r}_{i-1}\right))V(r_{i})A(q,r_{i})$$

where ${\rho (r}_{i})$ is the electron density described in the profile from Equation (1) at $r_{i}$. Thus, the form factor was calculated numerically from the electron density of the bilayer cross-section for a planar 2D system [93] but including the electron density profile described.

The thickness of the hydrocarbon core and of the hydrophilic layer of polar headgroups was estimated as $R_{hc}=2\sqrt{2\ln2}$ $\sigma_{hc}\;$ and $R_{ph}=2\sqrt{2\ln2}$ $\sigma_{ph}$, respectively. Thus, the total bilayer thickness was calculated as $T=2\sqrt{2\ln2}\left(2\sigma_{ph}+\sigma_{hc}\right)$.

**Scheme 1:**
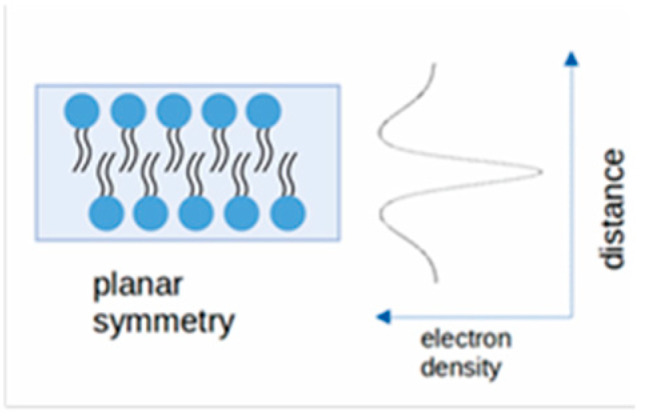
Electron density modeling of the bilayer.

In practice, the summation in the amplitude factor equation was performed using 100 steps in the range from −3σ to +3σ of the outer Gaussian. Therefore, the present modelling procedure could be used to estimate the symmetry of the supramolecular entity and the electron density distribution along an experimental axis, as shown in Scheme 1.

Generalized polarization and fluorescence anisotropy of Laurdan: the membrane order and fluidity of the TA-nanoARC and nanoARC were determined by measuring generalized polarization (GP) and fluorescence anisotropy (FA) using the environment-sensitive probe, Laurdan. Laurdan was incorporated into the nanovesicles at a molar ratio of Laurdan: PL of 1:500 (for GP) or 1:20 (for FA) in Tris-HCl buffer. The suspensions of labeled nanovesicles were incubated at room temperature for 30 min in the dark. GP was calculated using the following Equation:

$$GP=\frac{I_{440}-I_{490}}{I_{440}+I_{490}}$$

*I*_440_ and *I*_490_ represent the fluorescence intensities at λ = 440 nm and λ = 490 nm, respectively, obtained from the emission spectrum recorded between 400 and 520 nm using λ = 364 nm (excitation slit width: 5.0 nm; emission slit width: 10.0 nm; scan speed: 100 nm/min).

FA was calculated using the spectrofluorometer software according to the following Equation:

$$FA=\frac{I_{0}-G.I_{90}}{I_{0}+2G.I_{90}}$$

*I*_0_ and *I*_90_ represent the fluorescence intensities measured at λ = 440 nm with λ = 364 nm and the excitation polarizer oriented at 0° and 90°, respectively. The correction factor (G) was obtained from the ratio of emission intensities at 0° and 90° with the excitation polarizer set at 90°, after subtracting the contribution of scattered light.

### 4.6. Antioxidant Activity

#### 4.6.1. DPPH Assay

The antioxidant capacity of TA-nanoARC, nanoARC, and TA was evaluated by measuring DPPH•^+^ radical scavenging activity, following the method described by [94] with modifications. Briefly, 20 µL of TA-nanoARC (0–340 µg/mL PL), nanoARC, and TA (0–380 µg/mL PL) in methanol were mixed with 160 µL of a methanolic DPPH•^+^ solution in a 96-well plate and incubated at 37 °C for 30 min in the dark under agitation at 200 rpm. After incubation, absorbance was measured at 580 nm using a Cytation 5 Cell Imaging Multi-Mode Reader (BioTek Instruments, Winooski, VT, USA). BR exhibits strong absorption peaks at 490 nm, this is the reason why the measurements were performed at 580 nm instead of the conventional 517 nm [94]. The blank control contained DPPH•^+^ radical only. A calibration curve was constructed using Trolox (2.6–23 µg/mL). The assays were carried out with three independent experiments, each with three replicates.

#### 4.6.2. ABTS Assay

The ABTS radical cation (ABTS•^+^) was generated by mixing 500 µL of a 7 mM ABTS stock solution with 800 µL of 4 mM potassium persulfate and allowing the mixture to stand in the dark for 12–16 h before use. The ABTS•^+^ solution was then diluted with methanol to obtain an absorbance of 0.70 ± 0.02 at 734 nm, measured using a Metrolab M-330 spectrophotometer (Biodata) [95]. The samples, TA-nanoARC, nanoARC, and TA (0–150 µg/mL PL), were diluted in methanol, and 10 µL of each was mixed with 1 mL of the ABTS•^+^ solution. The mixture was incubated at 30 °C in the dark for 30 min, and absorbance was then measured at 734 nm. The blank control contained ABTS•^+^ radicals only. A Trolox calibration curve (0.6–6.2 µg/mL) was used for quantification. The assays were conducted in three independent experiments with three replicates each experiment.

For both the DPPH•^+^ and ABTS•^+^ assays, the percentage of radical inhibition was calculated using the following Equation:

$$Inhibition\;\%\;(I\%)=\frac{{Abs}_{blank}-{Abs}_{sample}}{{Abs}_{blank}}\times 100$$

where *Abs_blank_* refers to the absorbance of the control solution containing only the DPPH•^+^ or ABTS•^+^ radicals, and *Abs_sample_* corresponds to the absorbance of the sample incubated with the respective radical solution.

The resulting I% were plotted as a function of the PL concentrations of the samples. Subsequently, IC_50_ values of the samples were determined for each assay by fitting a nonlinear regression dose-response curve (Inhibitor vs. normalized response) using GraphPad Prism 8.0.1 (GraphPad Software, Inc., San Diego, CA, USA).

### 4.7. Storage Stability

The colloidal stability of TA-nanoARC and nanoARC was determined after 31 days of storage at 4 °C, protected from light in amber vials under atmosphere air. Particle size, pdi, and ζ potential were determined as previously described. Measurements were performed in three independent experiments (*n* = 3) with three replicates each. Stability was considered acceptable when variations in particle size, pdi, and ζ potential were not statistically significant as determined by the Wilcoxon signed-rank test using GraphPad Prism 8.0.1 (GraphPad Software, Inc., San Diego, CA, USA).

### 4.8. Stability upon Nebulization Process

The structural stability of nanoARC and TA-nanoARC upon nebulization using a vibrating mesh nebulizer (Omron NE-U22, OMRON Healthcare, Kyoto, Japan) was evaluated in terms of particle size, polydispersity index (Pdi), and percentage of PL recovery before (BN) and after nebulization (AN). Briefly, 2 mL of a fresh suspension of nanoARC or TA-nanoARC at 100 μg/mL PL were nebulized for 5 min, and the aerosol was collected in a flask connected to the nebulizer. Recovered lipids were assessed by quantifying PL content, and analyzing particle size and ζ potential, as previously described.

### 4.9. Cell Lines and Culture Conditions

Human lung epithelial cells (A549 cell line, ATCC^®^ CCL-185™), kindly provided by Dr. Boris Rodenak (Instituto de Investigaciones Bioquímicas de La Plata), were cultured in MEM supplemented with 10% FBS, 100 U/mL penicillin, 100 μg/mL streptomycin, and 2 mM L-glutamine.

Human monocytic cells (THP-1 cell line, ATCC^®^ TIB-202™), kindly provided by Dr. Jessica Minnaard (Centro de Investigación y Desarrollo en Criotecnología de Alimentos, CIDCA, National University of La Plata), were cultured in RPMI medium supplemented with 100 U/mL penicillin, 100 μg/mL streptomycin, 2 mM L-glutamine, 0.05 mM β mercaptoethanol, and 1 mM sodium pyruvate. Monocytes were differentiated into macrophages (mTHP-1) by treatment with 100 ng/mL PMA for 24 h. Both cell lines were incubated at 37 °C in a humidified atmosphere with 5% CO_2_.

### 4.10. Cell Viability

The viability of A549 and mTHP-1 cells after 48 h of incubation with nebulized TA-nanoARC, nanoARC, and m-T80 was assessed using the MTT assay, with slight modifications. Briefly, A549 and mTHP-1 cells were seeded in 96-well plates at densities of 1.1 × 10^4^ and 3.0 × 10^4^ cells per well, respectively, and cultured for 24 h. Cells were then incubated with the nebulized samples at PL concentrations ranging from 1.6 to 50 µg/mL and BR concentrations from 0.01 to 0.32 µg/mL.

After 48 h of incubation, the medium was removed, cells were washed with PBS, and 100 µL of 0.05% *w*/*v* MTT solution in medium was added to each well. Following a 2 h incubation at 37 °C and 5% CO_2_, the MTT solution was discarded, and the resulting formazan crystals were dissolved in 100 µL of dimethyl sulfoxide (DMSO) under orbital shaking at 100 rpm for 5 min. Absorbance was measured at 570 nm using a Cytation 5 multimode plate reader.

Cell viability was expressed as a percentage relative to untreated control cells, and IC_50_ values were calculated for each condition by applying a nonlinear regression dose–response curve model (Inhibitor vs. normalized response) using GraphPad Prism 8.0.1 (GraphPad Software, Inc., San Diego, CA, USA).

### 4.11. Effect on Lysosomes

The effect on lysosomes after uptake of TA-nanoARC and nanoARC by A549 cells was evaluated using the fluorescent acidotropic probe LysoTracker™ Red DND-99 (LR). Briefly, A549 cells were seeded at a density of 4.0 × 10^5^ cells per well in 24-well plates containing 12 mm round glass coverslips at the bottom and incubated for 24 h at 37 °C in a 5% CO_2_ atmosphere. Then, cells were then incubated with TA-nanoARC and nanoARC at non-cytotoxic (3.2 µg/mL PL) and cytotoxic (50 µg/mL PL) concentrations in MEM supplemented with 5% FBS for 24 h at 37 °C. After incubation, the supernatants were discarded, and cells were washed with PBS and incubated with 3.8 µM LR and 3.2 µM Hoechst for 30 min at 37 °C. Subsequently, cells were then washed five times with PBS and fixed with 3.75% *w*/*v* formaldehyde in PBS for 5 min. Finally, the preparations were incubated for 1 h at 37 °C and, after three washes with PBS, were mounted on microscope slides using 90% *w*/*v* glycerol mounting medium. Confocal microscopy images were acquired using a Leica TCS SP8 spectral laser scanning confocal microscope (Leica Microsystems, Wetzlar, Germany) with excitation/emission wavelengths of 577/590 nm for LR and 350/461 nm for Hoechst, respectively. The fluorescence intensity was quantified on 20× images as the media of 90 cells using Image J software 1.54K. Significant differences between formulations were determined using a one-way ANOVA followed by Dunnett’s test.

### 4.12. Effect on Mitochondrial Membrane Potential

The effect of TA-nanoARC and nanoARC on mitochondrial membrane potential in A549 cells was assessed using the JC-1 mitochondrial staining kit (Sigma-Aldrich, St. Louis, MO, USA), according to the manufacturer’s instructions. Briefly, A549 cells were seeded in 48-well plates at a density of 2 × 10^4^ cells per well and cultured for 24 h. Cells were then incubated with TA-nanoARC or nanoARC at concentrations of 3.2 µg/mL and 50 µg/mL of PL in MEM supplemented with 5% FBS. After 24 h of incubation, the supernatants were removed, and cells were washed with PBS and then incubated with 2.5 µg/mL of JC-1 staining solution for 20 min at 37 °C. Following incubation, the staining solution was discarded, cells were washed with PBS, and fluorescence intensity was measured using a Cytation™ 5 multimode plate reader. JC-1 monomer fluorescence was measured at λ_ex_ = 490 nm y λ_em_ = 530 nm, while JC-1 aggregate fluorescence was measured at λ_ex_ = 525 nm y λ_em_ = 590 nm. As a positive control, cells were treated with 100 ng/mL valinomycin solution [96].

### 4.13. Intracellular Anti-Reactive Oxygen Species Activity

The ability of TA-nanoARC or nanoARC to reduce reactive oxygen species (ROS) generation on LPS-stimulated mTHP-1 and A549 cells was measured using the carboxy-H_2_DCFDA probe. Briefly, A549 and mTHP-1 cells were seeded in 96-well plates at a density of 3 × 10^4^ cells per well and cultured for 24 h at 37 °C in a 5% CO_2_ incubator. Then, cells were co-incubated with 1 μg/mL of LPS and either TA-nanoARC or nanoARC (3.2 and 50 μg/mL PL) in fresh medium containing 5% FBS. After 24 h of incubation, adherent cells on the bottom of the well were washed twice with PBS and incubated with 4.0 µg/mL H_2_DCFDA solution for 30 min at 37 °C in the dark. LPS-stimulated cells without treatment were used as a reference control. Fluorescence intensity of the cells was measured using a Cytation 5 multimode reader (λ_ex_ 490 nm y λ_em_ 520 nm).

### 4.14. Immunomodulation of mTHP-1 Cells by A549 Cell-Conditioned Media

A549 cells were seeded at a density of 6 × 10^4^ cells per well in 24-well plates and incubated for 24 h. The cells were then treated with TA-nanoARC at a concentration of 6.30 µg/mL PL/0.04 µg/mL BR for 48 h. Simultaneously, mTHP-1 cells were seeded at a density of 2.1 × 10^5^ cells per well in 24-well plates using RPMI medium supplemented with 10% FBS. After A549 incubation, 320 µL of culture supernatants were transferred to each mTHP-1 well, along with 180 µL of RPMI medium supplemented with 5% FBS and 1% *w*/*v* sodium pyruvate. After 24 h of incubation, supernatants were collected and stored for subsequent analysis of proinflammatory cytokines. The mTHP-1 cells that remained adherent were washed with PBS, trypsinized, washed again, and stained with 20 µL of anti-CD204-APC antibody (0.25 mg/mL) for 30 min. Subsequently, cells were then washed with PBS and fixed with 4% *w*/*v* formaldehyde for 10 min at 4 °C. After 48 h, a total of 2000 cells were analyzed by flow cytometry using a 635 nm red diode laser and FL-4 filter on a FACSCalibur flow cytometer (Becton Dickinson, San Jose, CA, USA). Data were analyzed using FlowJo software version 10.8.0.

### 4.15. Determination of Pro-Inflammatory Cytokines in Human Macrophages

Human TNF-α, IL-8, and IL-6 concentrations were measured using the BD OptEIA™ enzyme-linked immunosorbent assay (ELISA) kits (BD Biosciences, San Jose, CA, USA) on supernatants collected from mTHP-1 cells, following the manufacturer’s instructions. Absorbance was read at 450 nm using the plate reader of the Cytation 5 system.

### 4.16. Statistical Analyses

Statistical analyses were performed using one-way ANOVA followed by Dunnett’s post hoc test with Prism 8.0 software (GraphPad, CA, USA). Statistical analyses for GP and FA, cell viability, lysosomes activity, anti-reactive oxygen species activity, mitochondrial membrane potential, and pro-inflammatory cytokines levels were also conducted using one-way ANOVA followed by Dunnett’s test, with significance considered at * *p* < 0.05 and ** *p* < 0.01 with Prism 8.0 software (GraphPad, CA, USA).

## Figures and Tables

**Table 1:** Structural features of nanovesicles. Values of Z average, polydispersity index (pdi), ζ potential, bacterioruberin/phospholipid (BR/PL), fluorescence anisotropy (FA), and generalized polarization (GP) are expressed as mean ± standard deviation (SD) (*n* = 16 TA-nanoARC; *n* = 6 nanoARC). Statistical significance of FA and GP compared to nanoARC was determined using a one-way ANOVA followed by Dunnett’s test, * *p* < 0.05; ** *p* < 0.01.

Formulation	Z Average (nm ± SD)	Pdi ± SD	ζ Potential (mV ± SD)	BR/PL (µg/mg ± SD)	FA	GP
TA-nanoARC	300.3 ± 84.2	0.59 ± 0.12	−20 ± 3.7	4.4 ± 1.8	0.18 ± 0.01 *	−0.11 ± 0.01 **
nanoARC	233.3 ± 65.0	0.61 ± 0.10	−24 ± 1.4	-	0.16 ± 0.01	−0.35 ± 0.04

**Table 2:** Core, hydrophilic layer, and bilayer thickness determined by fitting of SAXS data are expressed as mean ± standard deviation (SD), *n* = 3.

Formulation	Core (nm ± SD)	Hydrophilic Layer (nm ± SD)	Bilayer Thickness (nm ± SD)
TA-nanoARC	24.0 ± 2.0	14.0 ± 1.0	52.0 ± 4.0
nanoARC	25.4 ± 0.2	12.9 ± 0.2	51.2 ± 0.6

**Table 4:** Characterization of TA-nanoARC and nanoARC before (BN) and after nebulization (AN) in terms of Phospholipid recovery, Z average, polydispersity index (Pdi), and ζ potential expressed as mean ± standard deviation (SD), *n* = 3.

Formulation	TA-nanoARC		nanoARC	
Treatment	BN	AN	BN	AN
Phospholipid recovery (%)		83.3 ± 6.6		86.2 ± 3.3
Z average (nm ± SD)	254 ± 61	283 ± 122	306 ± 14	376 ± 83
Pdi ± SD	0.346 ± 0.121	0.827 ± 0.258	0.794 ± 0.299	0.434 ± 0.219
ζ Potential (mV ± SD)	−20.8 ± 4.0	−22.0 ± 4.6	−24.0 ± 4.0	−23.9 ± 4.6

**Table 5:** Inhibitory concentration 50% (IC_50_) after nebulization values of formulations on macrophages (mTHP-1) and lung epithelial cells (A549 cells) expressed as PL and bacterioruberin concentration (PL-BR).

Cells	Formulation	IC_50_ (µg/mL PL—µg/mL BR)
A549	TA-nanoARC	33.8–0.15
	nanoARC	49.6–0.0
mTHP-1	TA-nanoARC	64.7–0.3
	nanoARC	>50.0

**Table 3:** FTIR band assignments of TA-nanoAC, nanoARC, total archaeolipids (TA), neutral archaeolipids (NAs), and Tween 80.

Formulation	Stretching OH	CH_3_ Antisymmetric Stretching (2956 cm^−1^)	CH_2_ Antisymmetric Stretching (2924 cm^−1^)	CH_2_ Symmetric Stretching (2854 cm^−1^)	C=C Stretching Region (1600–1750)	C–O–C Eter Stretching Region: ~1100	Antisymmetric Stretching Vibration ν_as_ (PO_2_ 1220–1225 cm^−1^	C=O Stretching Vibrations
TA-nanoARC	-	2951	2922	2855		1055	1225	
nanoARC	-	2951 (70.4)	2924 (62.2)	2853 (68)		1061	1225	
TA	3294	2951 (69.8)	2920 (58.1)	2851 (71)		1097, 1049	1225	
NA	broad 3391	2953	2922	2853		1057	-	
Tween80	broad strong 3377					asymmetric C–O–C 1082	-	1739

## Data Availability

Data are contained within the article.
